# Effects of a Mediterranean Diet, Dairy, and Meat Products on Different Phenotypes of Dyslipidemia: A Preliminary Retrospective Analysis

**DOI:** 10.3390/nu13041161

**Published:** 2021-04-01

**Authors:** Elena Formisano, Andrea Pasta, Anna Laura Cremonini, Ilaria Di Lorenzo, Samir Giuseppe Sukkar, Livia Pisciotta

**Affiliations:** 1 Nutritional Unit ASL-1 Imperiese, Giovanni Borea Civil Hospital, 18038 Sanremo, Italy; formisano.elena@gmail.com; 2 Department of Internal Medicine, University of Genoa, 16132 Genoa, Italy; andreapasta93@gmail.com (A.P.); ilariadilorenzo25@libero.it (I.D.L.); 3 Dietetics and Clinical Nutrition Unit, IRCCS Policlinic Hospital San Martino, 16132 Genoa, Italy; annalauracremonini@gmail.com (A.L.C.); samir.sukkar@hsanmartino.it (S.G.S.)

**Keywords:** Mediterranean diet, saturated fatty acids, ASCVD prevention

## Abstract

Background: Dyslipidemia is one of the major causes of atherosclerotic cardiovascular disease (ASCVD) and a Mediterranean Diet (MD) is recommended for its prevention. The objectives of this study were to evaluate adherence to an MD at baseline and follow-up, in a cohort of dyslipidemic patients, and to evaluate how different food intakes can influence lipid profile, especially how different sources of saturated fatty acids impact lipid phenotype. Methods: A retrospective analysis was conducted on 106 dyslipidemic patients. Clinical characteristics, lipid profile, and food habits data were collected at baseline and after three months of follow-up with counseling. Adherence to an MD was evaluated with a validated food-frequency questionnaire (MEDI-LITE score). Results: The cross-sectional analysis showed that higher consumption of dairy products correlated independently with higher levels of total cholesterol (TC), high-density lipoprotein cholesterol (HDL-C), and low-density lipoprotein cholesterol (LDL-C) and with lower triglycerides (TG) levels. Instead, lower HDL-C and TG levels and higher TC levels were independently associated with higher consumption of meat products. Adherence to an MD significantly improved after the follow-up period, from a mean value of 10 ± 3 (median 10, IQR 8–12) to 13 ± 2 (median 14, IQR 12–15), *p* < 0.0001. Conclusions: Dyslipidemic patients benefit from counseling for improving their adherence to an MD. The high intake of dairy products was associated with less atherogenic hyperlipidemia, which was characterized by higher levels of TC and HDL-C as compared withs the intake of an excessive amount of meat products, which was associated with higher levels of TC and TG and lower levels of HDL-C.

## 1. Introduction

Dyslipidemia is a major cause of atherosclerotic cardiovascular disease (ASCVD) [1,2,3]. In particular, the most atherogenic form of dyslipidemia is associated with diabetes, insulin resistance conditions, and familial combined hypercholesterolemia, and it is characterized by elevated levels of low-density lipoprotein cholesterol (LDL-C) and triglycerides (TG), and low levels of high-density lipoprotein cholesterol (HDL-C) [4,5]. Considering the different risk factors for ASCVD, diet plays a key role [6]. A Mediterranean diet (MD) is the main dietary model recommended for the prevention of ASCVD [7] and is the reference diet model of the 2019 European Society of Cardiology/European Atherosclerosis Society (ESC/EAS) guidelines for the management of dyslipidemia [8]. In patients affected by hyperlipidemia, the MD recommends low intake of saturated fatty acids (SFAs), at least less than 10% of total energy intake (i.e., <7% in patients with hypercholesterolemia). Consequently, moderate restriction of milk and dairy product consumption should be balanced by a limited intake of meat and meat products [9], in particular, the preferred consumption of milk and dairy products should not exceed 180 g/day, while no more than 80 g/day of meat and meat products should be consumed [10]. Low-fat cheeses and semi-skimmed milk should be preferred for patients with dyslipidemia [11], and processed meats should not be recommended [12]. A high prevalence of plant-based food, such as whole grains, vegetables, and fruits are highly advisable according to the MD in order to reach a total amount of carbohydrates between 45 and 55% of total energy intake, and 25–40 g per day of total dietary fiber [8]. Furthermore, the MD encourages a moderate amount of seafood, regular consumption of olive oil, and increased physical activity [13,14]. A reduction in sugar intake and elimination of alcohol consumption is recommended for patients with hypertriglyceridemia [15].

In recent years, several studies have investigated the relationship between diet and ASCVD risk. Different studies have mostly recommended that consumption of SFAs is not recommended for prevention of ASCVD and increased LDL-C levels [16,17], while recent epidemiological studies in the literature support the fact that SFAs do not increase the risk of ASCVD [18]. The Prospective Urban and Rural Epidemiology study (PURE) was a large observational study that clarified the relationship between macronutrient intake and mortality, concluding that SFA intake did not influence mortality rate, while high carbohydrate intake was associated with higher mortality risk [19]. In the European Prospective Investigation into Cancer and Nutrition (EPIC) study, a significantly lower mortality was observed among subjects with the highest intake of saturated fatty acids as compared with those with minimum intake [20]. In a recent meta-analysis, de Souza et al. checked the relationship between SFAs intake and cardiovascular mortality and did not observe an increased risk of ASCVD events in subjects with a high consumption of SFAs as compared with those with low consumption [21]. Therefore, ASCVD risk may be influenced by the dietary source of SFAs, mainly represented by dairy and meat products. Meat consumption is considered to be a dietary risk factor for atherogenic dyslipidemia [12]. De Oliveira et al. reported, on the one hand, that a higher intake of SFAs from meat products is related to the development of ASCVD; on the other hand, a lower ASCVD risk is correlated to a higher intake of SFAs from dairy products [22]. However, the literature is still controversial regarding the relationship between meat and dairy products intake and alterations in lipid profile. Therefore, this study aims to evaluate adherence to an MD at baseline and at follow-up in a cohort of dyslipidemic patients and to evaluate how different food intakes can influence the lipid profile, especially how different sources of saturated fatty acids act on the lipid phenotype.

## 2. Materials and Methods

### 2.1. Study Design and Subjects

In the current study, a retrospective analysis was performed on the medical charts of 106 patients, 53 women and 53 men, suffering from different forms of hyperlipidemias. All subjects had been referred to the outpatient section of the Lipid Clinic, IRCCS Policlinic San Martino Hospital, University of Genoa, Italy, from February to July 2019. The exclusion criteria were age <18 years, active neoplasm, malignant hematological disease, endocrinopathy, inflammatory bowel disease, connective tissue disease, chronic and acute liver disease, congestive heart failure (NYHA class III–IV), acute and chronic nephropathy (GRF < 45 mL/min according to the Chronic Kidney Disease—Epidemiology Collaboration equation), acute and chronic infection, and therapy with hormones (including insulin) or with recombinant cytokines.

At baseline, height and weight, blood pressure, and smoking habits were recorded during a medical evaluation and body mass index (BMI) and the risk score (RS) were calculated. Blood test results provided a complete lipid profile (total cholesterol, high-density lipoprotein cholesterol, and triglycerides), tested without lipid lowering treatment and analyzed by an experienced physician who specialized in the management of hyperlipidemias. The LDL-C level calculation was performed using the Friedewald formula. Patients’ food habits at the time of the first evaluation (baseline) were assessed using a validated food frequency questionnaire, i.e., the MEDI-LITE score [10,23]. At the follow-up visit, i.e., after three months, weight and blood pressure were reported, as well as BMI and the blood lipid profile were recalculated; food habits were re-assessed using the same food frequency questionnaire, however, participants responded referring to the period between the baseline and the follow-up.

Informed written consent for the use of personal data was obtained from patients. The study was conducted in accordance with the Declaration of Helsinki and was approved by the Ethics Committee of IRCCS Policlinic Hospital San Martino in Genoa (Italy) (project number 44/2021).

### 2.2. Dietary Assessment

Food intake and adherence to an MD were assessed in common clinical practice through the MEDI-LITE score, which is a food frequency questionnaire that had been previously proposed and validated [10,24]. Daily and weekly food intake were evaluated by nine questions regarding foods recommended by the MD. The highest consumption of fruits, vegetables, cereals, legumes, fish, and olive oil corresponds to a score of 2, a score of 1 represents average consumption, and a score of 0 indicates the lowest consumption. Conversely, a score of 2 corresponds to the lowest consumption of meat and meat products, dairy products, and alcohol; a score of 1 indicates average intake; and a score of 0 represents the highest intake. The final score obtained ranged from 0 (low adherence to the MD) to 18 (high adherence to the MD) points.

#### Lifestyle Intervention

A lipid lowering diet based on the ESC/EAS guidelines for the management of dyslipidemias [8], was proposed to all patients. Lipid intake ranged between 25 and 35% of the daily kcal, with cholesterol lower than 300 mg/day and saturated fats <7% of the total kcal. The protein and carbohydrate intakes were 15–25% and 45–55% of the total daily kcal, respectively. Normal weight and overweight patients were advised to perform moderate-intense exercise at least 30 min per day. The following four different types of diets were administered, in an outpatient setting, after anthropometric and biochemical parameter evaluation:

- General advice with weekly counseling on food frequency for patients with primary hypercholesterolemia and normal weight patients.
- General advice with counseling on food frequency for non-overweight patients with mixed hyperlipemia or hypertriglyceridemia or with a reduction in alcohol and carbohydrates.
- General advice based on the frequency of food and control of food with an overall energy intake of 1700 kcal/day for overweight women.
- General advice based on the frequency of food and control of food portions with a total energy intake of 2100 kcal/day for overweight men.

The compositions of the diets are reported in Supplementary Table S1.

### 2.3. Statistical Analysis

Statistical analysis was performed using SPSS Statistics 25, Version 25.0 (SPSS Inc., Chicago, IL, USA) (https://www.ibm.com/it-it/analytics/spss-statistics-software, accessed on 31 March 2021). Detailed statistical analysis is reported in the Supplementary Materials.

## 3. Results

### 3.1. Characteristics of the Population and Adherence to an MD

The general characteristics of the study population at baseline are shown in Table 1. The median age was 55 (IQR 45–64) and the 106 patients were equally male and female. Most of them (*n* = 98, 92.5%) were natives of Liguria, a region in the North-West of Italy, while six patients were born in South America (5.7%) and one subject came from each of UK and Romania. Most of the enrolled patients (except two subjects) did not practice moderate physical activity for more than 15 min every day. The comedications used by patients are reported in Supplementary Table S2.

The mean MEDI-LITE score for the patients was 10 ± 3 (median 10, IQR 8–12) points. A regression analysis of the lipid profile adjusted for sex, age, BMI, and smoking habits showed that the presence of a higher MEDI-LITE score was independently correlated with higher levels of HDL-C levels (*β* ± SE 1.099 ± 0.413, r = 0.253, *p* = 0.009) and TC (*β* ± SE 1.353 ± 0.449, r = 0.283, *p* = 0.003) and lower levels of TG (*β* ± SE 3.712 ± 2.272, r = 159, *p* = 0.105). LDL-C levels did not correlate with the MEDI-LITE score.

### 3.2. Cross-Sectional Analysis: Relationship between Lipid Profile and Different Food Categories

Table 2 shows the number of patients based on the MEDI-LITE scores obtained for specific food categories. Lipid levels normalized by sex, age, BMI, and smoking habits were divided according to food categories and the score assigned (Table 2). Independent sample comparison tests were preliminarily performed. Patients with fruit intake >300 g/day had significantly higher levels of TC and HDL-C than those who ate <150 g/day or between 150 and 300 g/day. The levels of TC and HDL-C were significantly lower in subjects who ate fewer vegetables (<150 g/day) than in moderate and higher consumers of vegetables (150–300 and >300 g/day, respectively). No statistically significant differences were observed in the serum levels of LDL-C. Patients who consumed more meat and meat products (>120 g/day) had significantly lower levels of TC and HDL-C than moderate (80–120 g/day) and low (<80 g/day) meat consumers, while levels of TG were significantly higher in the latter (<80 g/day) than subjects who ate >120 g/day. LDL-C levels did not vary significantly. Patients who consumed the most (270 g/day) dairy products had significantly higher levels of TC, HDL-C, and LDL-C and lower levels of TG than patients with the lowest intake of dairy products (<180 g/day). No alcohol consumption was significantly associated with higher levels of TC and HDL-C and significantly lower levels of TG than alcohol consumption by patients. None differences in LDL-C levels have been observed in different alcohol consumer groups.

Finally, the relationships among the lipid profile (TC, HDL-C, LDL-C, and TG adjusted for sex, age, BMI, and smoking habits) and all food categories considered in the baseline analysis was investigated through a cross-sectional multivariate analysis (details of the statistical analysis are reported in Table 3. Higher consumption of dairy products correlated independently with higher levels of TC, HDL-C, and LDL-C and with a lower level of TG. Instead, lower levels of HDL-C and TG and higher levels of TC were independently associated with higher consumption of meat and meat products. Finally, a lower level of TC also correlated independently with the frequent use of olive oil. No other statistically significant differences were observed in LDL-C levels.

### 3.3. Follow-Up Analysis

Thirty-four patients (32.1%) did not attend the follow-up visit, and therefore were excluded from the follow-up analysis. Thus, demographical and clinical characteristics of the remaining 72 patients are reported in Table 4. The median follow-up period was 12 weeks (10–13 weeks).

Adherence to an MD significantly improved after the follow-up period, from a mean value of 10 ± 3 (median 10, IQR 8–12) to 13 ± 2 (median 14, IQR 12–15) with *p* < 0.0001 (Table 4). Overall, the number of patients with higher scores in the specific food categories considered in the MEDI-LITE score increased significantly, with the exception of olive oil and cereal consumption which did not statistically differ from baseline (Supplementary Table S3).

Nutritional counseling was effective for improving weight, BMI, and lipid profile excluding HDL-C levels. The addition of a nutraceutical or lipid-lowering drug was further effective in reducing TC, LDL-C, and TG levels (Table 5).

## 4. Discussion

The main purpose of this study was to evaluate the influence of different eating habits on the lipid profile of patients suffering from dyslipidemia.

A preliminary result is that greater adherence to an MD based on MEDI-LITE scores correlated with a better lipid profile characterized by higher levels of HDL-C and lower levels of TG, which is a finding that is strongly supported by the scientific literature [24]. Moreover, a recent study highlighted that high MEDI-LITE total scores were associated with low prevalence of dyslipidemia [25]. The results for fruits and vegetables intake showed an association with higher total cholesterol and HDL, but these data also indicate adherence to an MD and the effect on lipid profile may be mediated by dairy and olive oil consumption.

One of the main results of this study is the different impacts on the lipid profiles of patients with excessive consumption of meat and dairy products according to the MEDI-LITE scores. In fact, subjects with higher meat consumption had atherogenic dyslipidemia with significantly lower levels of HDL-C and higher levels of TG, while higher levels of TC and LDL-C were balanced by higher levels of HDL-C and lower levels of TG in patients with higher consumption of dairy products. These findings are questionable with respect to the dietary recommendations of the 2019 ESC/EAS guidelines for the management of dyslipidemia [8] which recommend an SFA intake less than 10% of the total caloric intake (i.e., about 22 g of SFAs considering a daily total caloric intake of 2000 kcal), and less than 7% in dyslipidemic patients, without distinguishing the food sources (i.e., meat or dairy products). In the literature, the effect of SFAs on ASCVD risk has been extensively studied but is not yet fully understood. Two large prospective analysis, the Nurses’ Health Study (NHS) [26] and the Health Professionals Follow-Up Study (HPFS) [27], on the one hand, reported that an increase in consumption of SFAs was related to increased risk of an ASCVD event [28]. On the other hand, a recent prospective study (PURE) clearly highlighted that the higher the consumption of SFAs, the lower the cardiovascular mortality, even in large consumers [19]. A similar correlation emerged in the EPIC study’s cohort of subjects, i.e., a minimum intake of SFAs was associated with significantly higher total mortality as compared with a maximum intake of SFAs [20]. A possible match point was proposed by the MESA study which prospectively observed a higher incidence of ASCVD events in patients who consumed more SFAs from meat, while SFAs from dairy products were associated with a decrease in ASCVD occurrence [22].

Furthermore, the correlation between ASCVD risk and higher meat consumption could also be due to the pro-atherogenic effect of some biomolecules in meat, such as choline, carnitine, and lecithin. Conversely, dairy products provide micronutrients and vitamins with a proven protective effect on the risk of ASCVD. In addition, Lordan et al. [29] highlighted the anti-inflammatory properties of dairy products because of their content in inhibitors of the platelet activating factor (PAF). The latter biomolecule is a lipoid factor of thrombosis and inflammation and plays a pivotal role in atherogenesis and atherosclerosis progression. To date, the protective effect of PAF inhibitors present in dairy products has been confirmed in vitro [30] and in vivo in both animals and humans [31]. Furthermore, beneficial anti-inflammatory properties for fermented dairy products have been hypothesized due to the presence of specific bacteria such as lactic acid bacteria and bifidobacteria, as well as the presence of specific fermentation products [32].

In brief, the source of SFAs could have different impacts on the ASCVD risk, in fact, most of the correlation studies between ASCVD and SFAs conducted in the USA, of a population consuming large quantities of meat products [33,34], have shown an increase in ASCVD risk proportional to the consumption of SFAs [26,27]. Conversely, the latter correlation between SFA consumption and ASCVD risk is negative in European patients [20] whose prevalent source of SFAs is represented by dairy products [35].

Moreover, the cross-sectional analysis highlights that frequent use of olive oil correlates with lower levels of TC; a meta-analysis by George, E. S. et al. reported that TC levels decreased linearly with high consumption of polyphenols olive oil [36].

The follow-up analysis showed that adherence to an MD and lipid profile levels improved with dietary counseling. In fact, it is known that nutritional counseling improves adherence to an MD, as highlighted in a recent study by Sialvera, T.E. et al., in which a positive change in lipid profile levels was also observed [37]. Overall, we observed a statistically significant shift from the categories with the lowest MEDI-LITE scores to those with the highest scores, except for olive oil and cereals, whose consumption was already high at the baseline. The reduction in dietary intake of SFAs has mostly been encouraged in accordance with current ESC/EAS guideline recommendations [8]. However, the reduction in meat products was preferred, and the categories of both high and medium consumption were reduced. Conversely, moderate consumption of dairy products was encouraged despite the high and low consumption categories. Further research and scientific debate will be needed to adapt the correct dietary recommendations to the results of the recent scientific literature [38].

Furthermore, dietary counseling was effective in reducing BMI, and the efficacy of dietary intervention in the treatment of weight is well known in the literature [39]. The use of lipid-lowering nutraceuticals had a valuable impact on the lipid profile as compared with diet alone and their effects have been previously highlighted in the literature [14,40,41].

The main limitation of the present study was the relatively small sample size analyzed; thus, the findings should be considered as preliminary. Other limitations are the lack of information about physical activity, employment, and family income; however, these indicators could be homogeneous as most patients live in the same local geographic area. Finally, the use of a food frequency questionnaire may be subject to recall bias.

## 5. Conclusions

In conclusion, high intake of dairy products was associated with a balanced hyperlipidemia, characterized by higher levels of TC and HDL-C, while a diet with an excessive amount of meat products caused a form of mixed atherogenic dyslipidemia with higher TC and TG levels and lower HDL-C levels. In the light of these findings and according to the recent literature, dietary recommendations should distinguish between SFA sources (i.e., meat products or dairy products) rather than suggesting a general reduction in SFA intake. In addition, dietary counseling is effective in improving adherence to MD in dyslipidemic patients.

## Figures and Tables

**Table 1:** Characteristics of all 106 dyslipidemic patients.

VARIABLE	VALUE
Sex [F/M: *n*; %]	53 (50.0%)/53 (50.0%)
Age [years: mean ± SD; median; IQR]	54 ± 14; 55 (45–64)
Weight [kg: mean ± SD; median; IQR]	73.9 ± 17.3; 72.5 (60.0–84.0)
BMI [kg/m^2^: mean ± SD; median; IQR]	26.1 ± 4.7; 25.8 (22.6–29.2)
SBP [mm/Hg: mean ± SD; median; IQR]	137 ± 17; 135 (128–146)
DBP [mm/Hg: mean ± SD; median; IQR]	82 ± 9; 80 (77–88)
Smoking habits [Never + Past/Current: *n*; %]	84 (79.2%)/22 (20.8%)
Risk SCORE [%: mean(SD; median; IQR]	4.0 ± 6.3; 1.4 (0.6–4.2)
Low-Risk: <1% [*n*; %]	41 (38.7%)
Moderate-Risk: ≥1% and <5% [*n*; %]	41 (38.7%)
High-Risk: ≥5% and <10% [*n*; %]	10 (9.4%)
Very-High-Risk: ≥10% [*n*; %]	14 (13.2%)
TC [mg/dl: mean ± SD; median, IQR]	245 ± 55; 248 (210–278)
HDL-C [mg/dl: mean ± SD; median, IQR]	57 ± 19; 53 (42–66)
LDL-C [mg/dl: mean ± SD; median, IQR]	159 ± 51; 154 (130–189)
TG [mg/dl: mean ± SD; median, IQR]	186 ± 156; 127 (97–208)

Abbreviations: M = male, F = female, BMI = body mass index, SBP = systolic blood pressure, DBP = diastolic blood pressure, IQR= Interquartile range, TC = total cholesterol, HDL-C = high-density lipoprotein cholesterol, LDL-C = low-density lipoprotein cholesterol, TG = triglycerides.

**Table 2:** Lipid profile and patient distribution according to food categories and the score assigned.

	Patients [*n*, %]	TC [Median, IQR]	HDL-C [Median, IQR]	LDL-C [Median, IQR]	TG [Median, IQR]
**Fruit**					
<150 g/day	26 (24.5%)	238 (229–250)	52 (42–58)	156 (148–164)	220 (142–270)
150–300 g/day	20 (18.9%)	238 (232–247)	54 (48–66)	161 (147–164)	209 (132–227)
>300 g/day	60 (56.6%)	251 (239–259)	59 (51–68)	163 (150–165)	149 (134–225)
*p*-value †		*p* = 0.003 <150 vs. >300 g/day *p* = 0.01 (*p* = 0.003) 150–300 vs. >300 g/day *p* = 0.04 (*p* = 0.01)	*p* = 0.03 <150 vs. >300 g/day *p* = 0.04 (*p* = 0.01)	NS	NS
**Vegetables**					
<100 g/day	36 (34.0%)	237 (230–249)	51 (45–60)	155 (149–164)	217 (145–235)
100–250 g/day	32 (30.2%)	249 (238–257)	57 (51–70)	163 (155–164)	145 (126–222)
>250 g/day	38 (35.8%)	249 (238–259)	57 (52–68)	164 (149–166)	149 (137–217)
*p*-value †		*p* = 0.01 <100 vs. >250 g/day *p* = 0.02 (*p* = 0.008) <100 vs. 100–250 g/day *p* = 0.06 (*p* = 0.02)	*p* = 0.04 <100 vs. >250 g/day *p* = 0.10 (*p* = 0.03) <100 vs. 100–250 g/day *p* = 0.06 (*p* = 0.02)	NS	NS
**Legumes**					
<70 g/week	50 (47.2%)	245 (233–255)	55 (49–67)	162 (149–164)	157 (134–229)
70–140 g/week	46 (43.4%)	246 (236–259)	56 (50–68)	164 (150–165)	149 (148–163)
>140 g/week	10 (9.4%)	239 (237–254)	56 (49–61)	157 (128–227)	216 (153–231)
*p*-value †		NS	NS	NS	NS
**Cereals**					
<130 g/day	40 (37.7%)	247 (233–259)	57 (50–68)	162 (149–165)	150 (130–230)
130–200 g/day	20 (18.9%)	244 (237–256)	58 (50–64)	163 (155–165)	180 (137–230)
>200 g/day	46 (43.4%)	243 (235–253)	55 (49–62)	163 (149–165)	198 (139–227)
*p*-value †		NS	NS	NS	NS
**Fish**					
<100 g/week	32 (30.2%)	240 (232–252)	55 (45–64)	161 (149–164)	212 (125–252)
100–250 g/week	58 (54.7%)	248 (236–258)	59 (50–67)	164 (149–165)	150 (133–220)
>250 g/week	16 (15.1%)	241 (237–254)	54 (50–63)	150 (149–165)	209 (150–231)
*p*-value †		NS	NS	NS	NS
**Meat Products**					
>120 g/day	21 (19.8%)	236 (228–249)	48 (40–56)	158 (148–165)	228 (169–270)
80–120 g/day	31 (29.2%)	240 (233–249)	53 (49–61)	155 (148–164)	214 (139–230)
<80 g/day	54 (50.9%)	253 (240–259)	60 (52–69)	164 (156–165)	147 (127–212)
*p*-value †		*p* = 0.001 <80 vs. >120 g/day *p* = 0.001 (*p* < 0.0001) <80 vs. 80–120 g/day *p* = 0.06 (*p* = 0.02)	*p* < 0.0001 <80 vs. 80–120 g/day *p* = 0.06 (*p* = 0.02) <80 vs. >120 g/day *p* < 0.0001 (*p* < 0.0001)	NS	*p* = 0.008 <80 vs. >120 g/day *p* = 0.009 (*p* = 0.003)
**Dairy Products**					
>270 g/day	51 (48.1%)	249 (240–259)	58 (52–68)	164 (150–166)	145 (127–216)
180–270 g/day	11 (10.4%)	237 (231–255)	53 (42–61)	161 (152–165)	219 (149–240)
<180 g/day	44 (41.5%)	240 (233–253)	53 (47–66)	156 (148–164)	211 (138–258)
*p*-value †		*p* = 0.01 <180 vs. >270 g/day *p* = 0.02 (*p* = 0.005)	*p* = 0.074 <180 vs. >270 g/day *p* = 0.04	*p* = 0.02 <180 vs. >270 g/day *p* = 0.02 (*p* = 0.006)	*p* = 0.02 <180 vs. >270 g/day *p* = 0.04 (*p* = 0.01)
**Alcohol**					
>2 AU/day	35 (33.0%)	239 (233–254)	54 (47–67)	157 (149–164)	213 (147–234)
1–2 AU/day	34 (32.1%)	241 (234–252)	53 (48–60)	161 (149–164)	180 (124–236)
<1 AU/day	37 (34.9%)	253 (242–260)	59 (54–68)	164 (150–165)	146 (134–213)
*p*-value †		*p* = 0.009 1–2 vs. <1 AU *p* = 0.02 (*p* = 0.006) >2 vs. <1 AU *p* = 0.04 (*p* = 0.01)	*p* = 0.01 1–2 vs. <1 AU *p* = 0.05 (*p* = 0.02) >2 vs. <1 AU *p* = 0.12 (*p* = 0.04)	NS	>2 vs. <1 AU *p* = 0.01
**Olive Oil**					
Occasional	3 (2.8%)	255 (233–259)	60 (43–63)	166 (166–166)	150 (149–234)
Frequent	5 (4.7%)	247 (245–248)	59 (51–71)	163 (155–169)	135 (106–230)
Regular	98 (92.5%)	243 (235–256)	56 (49–67)	162 (149–165)	191 (134–228)
*p*-value †		NS	NS	NS	NS

Abbreviations: AU = Alcoholic Unit; NS = Non-statistically significant. † Independent samples Kruskal–Wallis tests. Significance values have been adjusted by the Bonferroni correction for multiple tests. Unadjusted *p*-values have been also reported.

**Table 3:** Multivariate analysis on baseline lipid profile in all 106 patients.

VARIABLE and PREDICTORS	*β*	SE	*p*-Value	r^2^	F (*p*-Value) †
**TC**				0.317	4.952 (<0.0001)
Fruit (high intake: >300 g/day)	2.373	1.434	0.101		
Vegetables (high intake: >250 g/day)	2.628	1.429	0.069		
Legumes (high intake: >140 g/day)	1.292	1.726	0.456		
Cereals (high intake: >200 g/day)	−0.387	1.186	0.745		
Fish (high intake: >250 g/day)	0.594	1.830	0.746		
Meat products (low intake: <80 g/day)	4.784	1.408	0.001		
Dairy Products (low intake: <180 g/day)	−2.596	1.160	0.028		
Olive Oil (Frequent use)	−5.495	2.868	0.058		
Alcohol (low intake: < 1 AU/day)	2.082	1.340	0.124		
**HDL-C**				0.268	3.904 (<0.0001)
Fruit (high intake: >300 g/day)	1.791	1.353	0.189		
Vegetables (high intake: >250 g/day)	1.626	1.347	0.230		
Legumes (high intake: >140 g/day)	1.53	1.627	0.350		
Cereals (high intake: >200 g/day)	−0.328	1.118	0.770		
Fish (high intake: >250 g/day)	−0.766	1.726	0.658		
Meat products (low intake: <80 g/day)	5.359	1.328	<0.0001		
Dairy Products (low intake: <180 g/day)	−2.433	1.094	0.048		
Olive Oil (Frequent use)	−2.643	2.704	0.331		
Alcohol (low intake: < 1 AU/day)	1.034	1.264	0.416		
**LDL-C**				0.149	1.540 (0.149)
Fruit (high intake: >300 g/day)	0.406	1.187	0.733		
Vegetables (high intake: >250 g/day)	1.700	1.229	0.171		
Legumes (high intake: >140 g/day)	0.301	1.515	0.843		
Cereals (high intake: >200 g/day)	0.038	1.013	0.970		
Fish (high intake: >250 g/day)	−0.602	1.589	0.706		
Meat products (low intake: <80 g/day)	1.186	1.246	0.344		
Dairy Products (low intake: <180 g/day)	−2.190	0.976	0.028		
Olive Oil (Frequent use)	−4.877	2.663	0.071		
Alcohol (low intake: < 1 AU/day)	0.840	1.185	0.481		
**TG**				0.233	3.202 (0.002)
Fruit (high intake: >300 g/day)	−6.806	7.468	0.364		
Vegetables (high intake: >250 g/day)	−9.251	7.474	0.219		
Legumes (high intake: >140 g/day)	−0.479	9.005	0.958		
Cereals (high intake: >200 g/day)	3.714	6.225	0.552		
Fish (high intake: >250 g/day)	0.955	9.530	0.920		
Meat products (low intake: <80 g/day)	−19.321	7.358	0.010		
Dairy Products (low intake: <180 g/day)	15.326	6.065	0.013		
Alcohol (low intake: < 1 AU/day)	−9.931	7.080	0.164		
Olive Oil (Frequent use)	17.823	14.928	0.235		

Abbreviations: TC = total cholesterol, HDL-C = high-density lipoprotein cholesterol, LDL-C = low-density lipoprotein cholesterol, TG = triglycerides. Dependent variable were TC, HDL-C, LDL-C and TG (bold text) and were adjusted for sex, age, BMI and smoking habits. Predictors were fruit intake (<150 g/day = 0, 150–300 g/day = 1 and >300 g/day = 2), vegetables intake (<100 g/day = 0, 100–250 g/day =1 and >250 g/day = 2), legumes intake (<70 g/week = 0, 70–140 g/week = 1 and >140 g/week = 2), cereals intake (<130 g/day = 0, 130–200 g/day = 1 and >200 g/day = 2), fish intake (<100 g/week = 0, 100–250 g/week = 1 and >250 g/week = 2), meat products intake (>120 g/day = 0, 80–120 g/day = 1 and <80 g/day = 2), dairy products intake (>270 g/day = 0, 180–270 g/day = 1 and <180 g/day = 2), alcohol consume (>2 AU/day = 0, 1–2 AU/day = 1 and <1 AU/day = 2) and olive oil use (Occasional = 0, Frequent = 1 and Regular = 2). Abbreviation: *β* = angular coefficient, SE = standard error, r^2^ = square correlation coefficient, F = F-value, *p*-values for predictors, † *p*-value for model fitting significance.

**Table 4:** Characteristics of the 72 dyslipidemic patients included in follow-up analysis.

VARIABLE	VALUE
Sex [F/M: *n*; %]	34 (47.2%)/38 (52.8%)
Age [years: mean ± SD; median; IQR]	55 ± 13; 55 (48–64)
SBP [mm/Hg: mean ± SD; median; IQR]	138 ± 17; 136 (130–150)
DBP [mm/Hg: mean ± SD; median; IQR]	83 ± 10; 81 (78–89)
Smoking habits [Never + Past/Current: *n*; %]	55 (76.4%)/17 (23.6%)
Risk SCORE [%: mean(SD; median; IQR]	4.3 ± 7.0; 1.5 (0.7–4.1)
Low-Risk: <1% [*n*; %]	25 (34.7%)
Moderate-Risk: ≥1% and <5% [*n*; %]	31 (43.1%)
High-Risk: ≥5% and <10% [*n*; %]	6 (8.3%)
Very-High-Risk: ≥10% [*n*; %]	10 (13.9%)
Lipid Lowering Intervention	
Diet alone [*n*; %]	31 (43.1%)
Lipid-lowering Nutraceuticals [*n*; %]	13 (18.1%)
Lipid-lowering Drugs [*n*; %]	28 (38.9%)

Abbreviations: M = male, F = female, IQR = Interquartile range, SBP = systolic blood pressure, DBP = diastolic blood pressure.

**Table 5:** Variation in anthropometric measures, MEDI-LITE score, and lipid profile after the nutritional counseling.

VARIABLES	Baseline [Mean ± SD; Median; IQR]	Follow-up [Mean ± SD; Median; IQR]	Absolute Variation [Mean ± SD; Median; IQR]	Percentage Variation [%]	*p*-Value †
Weight [kg: mean ± SD; median; IQR]	75.7 ± 17.5; 74.3 (62.5, 84.0)	72.8 ± 15.8; 71.0 (60.0, 83.5)	−2.5 ± 3.5; −2.0 (−3.3, 0)	−3.2%	<0.0001
BMI [kg/m^2^: mean ± SD; median; IQR]	26.3 ± 4.7; 26.0 (22.9, 28.8)	25.2 ± 4.0; 25.5 (22.1, 27.4)	−9 ± 1.2; −0.6 (−1.2, 0)	−3.3%	<0.0001
MEDI-LITE [Points: mean ± SD; median; IQ range]	10 ± 3; 10 (8, 12)	13 ± 2; 14 (12, 15)	3 ± 3; 3 (1, 5)	+43.4%	<0.0001
TC [mg/dl: mean ± SD; median, IQR]					
Diet alone	249 ± 36; 255 (222, 267)	207 ± 54; 204 (158, 248)	−42 ± 54; −23 (−87, −1)	−16.2%	0.002
Lipid-lowering Nutraceuticals	257 ± 39; 261 (232, 270)	211 ± 39; 204 (190, 213)	−38 ± 34; −42 (−67, 0)	−15.1%	0.046
Lipid-lowering Drugs	238 ± 67; 234 (179, 294)	170 ± 30; 173 (140, 196)	−83 ± 61; −73 (−119, −33)	−29.3%	<0.0001
HDL-C [mg/dl: mean ± SD; median, IQR]					
Diet alone	58 ± 21; 53 (42, 67)	60 ± 20; 54 (47, 68)	0 ± 7; 0 (−3, 4)	2.5%	0.641
Lipid-lowering Nutraceuticals	58 ± 26; 46 (40, 63)	55 ± 17; 50 (40, 72)	0 ± 9; 0 (−2, 7)	3.4%	0.753
Lipid-lowering Drugs	54 ± 15; 52 (42, 66)	50 ± 12; 48 (40, 61)	−2 ± 8; −3 (−5, 4)	−2.5%	0.383
LDL-C [mg/dl: mean ± SD; median, IQR]					
Diet alone	161 ± 35; 150 (138, 187)	123 ± 47; 129 (83, 170)	−32 ± 49; −22 (−78, 0)	−18.6%	0.026
Lipid-lowering Nutraceuticals	180 ± 33; 179 (154, 196)	132 ± 34; 131 (102, 145)	−39 ± 37; −39 (−52, −9)	−22.6%	0.068
Lipid-lowering Drugs	146 ± 60; 147 (95, 185)	90 ± 29; 100 (70, 112)	−71 ± 50; −66 (−111, −22)	−38.3%	0.001
TG [mg/dl: mean ± SD; median, IQR]					
Diet alone	184 ± 123; 130 (103, 254)	129 ± 66; 111 (85, 167)	−39 ± 83; −17 (−44, 0)	−15.2%	0.025
Lipid-lowering Nutraceuticals	192 ± 149; 119 (102, 208)	125 ± 56; 102 (90, 177)	−54 ± 104; −10 (−81, 0)	−16.8%	0.173
Lipid-lowering Drugs	218 ± 228; 138 (104, 206)	152 ± 105; 123 (90, 158)	−80 ± 200; −45 (−63, 0)	−16.7%	0.013

Abbreviations: BMI = body mass index, IQR= Interquartile range. † *p*-values for dependent samples nonparametric Wilcoxon Signed Ranks Test between baseline and follow-up values.

## Data Availability

The data presented in this study are available on request from the corresponding author. The data are not publicly available due to privacy policy.

## Supplementary Materials

The following are available online at https://www.mdpi.com/article/10.3390/nu13041161/s1: Supplementary Table S1, Composition of diets for overweight patients; 1.1 General advice with counselling on weekly food frequency for patients with primary hypercholesterolemia and normal weight patients; 1.2 General advice with advising on food frequency in non-overweight patients with mixed hyperlipemia or hypertriglyceridemia or with reduction in alcohol and carbohydrates; 1.3 General advice based on frequency of food and control of food with an overall energy intake of 1700 kcal/day for overweight women; 1.4 General advice based on frequency of food and control of food portions with a total energy intake of 2100 kcal/day for overweight men; 1.5 Statistical Analysis; Supplementary Table S2, Comedications used by the 106 patients; Supplementary Table S3, Variation of patient distribution according to food categories and the score assigned.
