# Supplementary material for: Effects of a Mediterranean Diet, Dairy, and Meat Products on Different Phenotypes of Dyslipidemia: A Preliminary Retrospective Analysis
Source: Nutrients. 2021 Apr 1;13(4):1161. doi: 10.3390/nu13041161 (PMC8065939; doi:10.3390/nu13041161)
Supplement: Supplementary file 1 [file nutrients-13-01161-s001.pdf]

Supplementary Material Index

Supplementary Table S1. Composition of diets for overweight patients.. 2

1.1 General advice with counselling on weekly food frequency for patients with primary hypercholesterolemia and normal weight patients 3

1.2 General advice with advising on food frequency in non-overweight patients with mixed hyperlipemia or hypertriglyceridemia or with reduction of alcohol and carbohydrates... 4

1.3 General advice based on frequency of food and control of food with an overall energy intake of 1700 kcal / day for overweight women 5

1.4 General advice based on frequency of food and control of food portions with a total energy intake of 2100 kcal / day for overweight men 6

1.5 Statistical analysis 8

Supplementary Table S2. Comedications used by the 106 patients. 9

Supplementary Table S3. Variation of patient distribution according to food categories and the MEDI-LITE score assigned 10

Supplementary Table S1. Composition of diets for overweight patients.

**NUTRIENTS**	1700 Kcal diet for females	2100 Kcal diet for males
**Proteins** [g, % of total daily energy intake]	72.7 (17.4%)	92.3 (17.8%)
**Fats** [g, % of total daily energy intake]	58.6 (31.5%)	72.9 (31.5 %)
**Carbohydrates** [g, % of total daily energy intake]	213.5 (51.1%)	263.5 (50.7%)
**Sugars** [g/day]	61.4	65.7
**Fiber** [g/day]	24.0	26.2
**Cholesterol** [mg/day]	136.9	159.5
**Saturated Fatty acids** [g, % of total daily energy intake]	10.2 (5.5%)	12.4 (5.4%)

1.1 General advice with counselling on weekly food frequency for patients with primary hypercholesterolemia and normal weight patients

1. Limit meat consumption twice a week and prefer white meat (i.e. poultry or lean pork), deprived of skin and visible fat. Limit red meat consumption once a week. Avoid offal consumption (i.e. brain and kidney).
2. Increase fish products consumption at least 2-4 times a week; prefer fish rich in omega-3 fatty acids (sardines, herring, anchovies, salmon). Crustaceans and mollusks are not contraindicated because they contain moderate amounts of cholesterol which are lower than meat and cheese.
3. Limit processed meat consumption once a week and prefer hams deprived of visible fat and turkey. Avoid sausages, bacon, salami and corned beef consumption.
4. Limit low-fat cheeses and dairy products consumption twice a week. Grated Parmesan should be limited to 1 tablespoon per day.
5. We recommend daily use of partially skimmed or skimmed milk and yogurt.
6. Limit eggs consumption once a week.
7. Avoid butter, margarine, lard, palm oil and foods that contain them. Use daily extra virgin olive oil daily. Avoid also fried foods, mayonnaise and pre-cooked frozen food.
8. Eat daily bread, pasta, rice, or other cereals, preferably whole meal. Avoid consumption of fresh egg pasta, oil or milk bread and focaccia.
9. Consume legumes at least 2-3 times a week (beans, chickpeas, lentils, peas, broad beans).
10. Consume vegetables and vegetables of any kind daily. Potatoes are not considered vegetables; they are substitutes of bread and pasta and must be consumed no more than once a week due to the high glycemic index.
11. Consume fruit daily. Limit frequency of consumption of very ripe and sugary fruits (i.e. bananas, persimmons, figs, prickly pears, grapes). Avoid coconut for saturated fat content.
12. Consume a small portion of dried nuts daily (walnuts, almonds, hazelnuts, cashews, pistachios).
13. Limit simple sugars consumption at breakfast (i.e. sugar, honey, jam). Limit sugar to 2 teaspoons per day. Avoid biscuits, ice cream, pastry desserts and milk or white chocolate. Without a calorie limit, it is possible to consume homemade desserts occasionally, prepared with extra virgin olive oil, yogurt or partially skimmed milk. Extra dark chocolate is allowed in limited quantities.
14. Alcohol consumption is not recommended. Avoid sugar drinks, industrial teas and fruit juices with or without added sugar.
15. 2-3 cups of coffee per day are allowed.
16. Recommended cooking methods: grilled, grilled, steamed, boiled, roasted, in foil, in the oven. To season the dishes, we recommend to use vegetable or meat broth, tomato sauce, vegetables, spices such as basil, oregano, saffron, marjoram, pepper, garlic.
17. Limit use of salt and avoid all foods rich in salt (pickled or canned foods).
18. It is recommended to do regular moderate-intensity exercise (≥ 30 minutes a day)

1.2 General advice with advising on food frequency in non-overweight patients with mixed hyperlipemia or hypertriglyceridemia or with reduction of alcohol and carbohydrate

1. Limit meat consumption twice a week and prefer white meat (i.e. poultry or lean pork), deprived of skin and visible fat. Limit red meat consumption once a week. Avoid offal consumption (i.e. brain and kidney).
2. Increase fish products consumption at least 2-4 times a week; prefer fish rich in omega-3 fatty acids (sardines, herring, anchovies, salmon). Crustaceans and mollusks are not contraindicated because they contain moderate amounts of cholesterol which are lower than meat and cheese.
3. Limit processed meat consumption once a week and prefer hams deprived of visible fat and turkey. Avoid sausages, bacon, salami and corned beef consumption.
4. Limit low-fat cheeses and dairy products consumption twice a week. Grated Parmesan should be limited to 1 tablespoon per day.
5. We recommend daily use of partially skimmed or skimmed milk and yogurt.
6. Limit eggs consumption once a week.
7. Avoid butter, margarine, lard, palm oil and foods that contain them. Use daily extra virgin olive oil daily. Avoid also fried foods, mayonnaise, and pre-cooked frozen food.
8. Eat daily bread, pasta, rice, or other cereals, preferably whole meal. Avoid consumption of fresh egg pasta, oil or milk bread and focaccia.
   Bread portion: 100 g per day (or wholemeal bread: 130 g per day) pasta portion: 80 g per day.
9. Consume legumes at least 2-3 times a week (beans, chickpeas, lentils, peas, broad beans).
10. Consume vegetables and vegetables of any kind daily. Potatoes are not considered vegetables; they are substitutes of bread and pasta and must be consumed no more than once a week due to the high glycemic index. Vegetable portion: free. Potato portion: 350 g instead of 80 g of pasta or 100 g of bread.
11. Consume fruit daily. Limit frequency of consumption of very ripe and sugary fruits (i.e. bananas, persimmons, figs, prickly pears, grapes). Avoid coconut for saturated fat content.
    Fruit portion: two per day.
    Portion of pineapple oranges, cherries, kiwi, mapo, pears, apples, plums: 150 g
    Portion of apricots, watermelon, strawberries, raspberries, quinces, melon, blueberries, blackberries, medlars, peaches, grapefruit: 210 g
    Fruits to be limited: bananas, persimmons, figs, prickly pears, mandarins, mangoes, pomegranates, grapes: 100 g
12. Consume a small portion of dried nuts daily (walnuts, almonds, hazelnuts, cashews, pistachios). Portion: 20 g
13. Avoid ice creams, pastry desserts and chocolate consumption. Eat rusks or simple biscuits for breakfast. Sugar should be avoided.
14. Alcohol consumption is not recommended. Avoid sugar drinks, industrial teas and fruit juices with or without added sugar.
15. 2-3 cups of coffee per day are allowed.
16. Recommended cooking methods: grilled, grilled, steamed, boiled, roasted, in foil, in the oven. To season the dishes, we recommend to use vegetable or meat broth, tomato sauce, vegetables, spices such as basil, oregano, saffron, marjoram, pepper, garlic.
17. Limit use of salt and avoid all foods rich in salt (pickled or canned foods).
18. It is recommended to do regular moderate-intensity exercise (≥ 30 minutes a day)

1.3 General advice based on frequency of food and control of food with an overall energy intake of 1700 kcal / day for overweight women

1. Limit meat consumption twice a week and prefer white meat (i.e. poultry or lean pork), deprived of skin and visible fat. Limit red meat consumption once a week. Avoid offal consumption (i.e. brain and kidney).
   Portion: 100 g
2. Increase fish products consumption at least 2-4 times a week; prefer fish rich in omega-3 fatty acids (sardines, herring, anchovies, salmon). Crustaceans and mollusks are not contraindicated because they contain moderate amounts of cholesterol which are lower than meat and cheese.

	**Portion**
Lobster, crab, shrimp, mussel, squid, cuttlefish, oyster, boga, octopus, sea snails, anchovy, cod, hake, soaked cod, stockfish, sea bream, sole, murmur, snapper, grouper, corvina, dogfish, trout, pagro , croaker, moray, bass, breed, turbot, anglerfish, tench, pike.	150 g
fresh salmon, fresh tuna, carp, mullet, swordfish, mackerel, sardine, herring, sturgeon, sarago, verdesca, latterini, eel.	100 g

3. Limit processed meat consumption once a week and prefer hams deprived of visible fat and turkey. Avoid sausages, bacon, salami and corned beef consumption.
   Portion: raw ham 90 g or
   cooked ham 100 g or
   turkey 120 g.
4. Limit low-fat cheeses and dairy products consumption twice a week. Grated Parmesan should be limited to 1 tablespoon per day.
   Low-fat cheese portion: 50 g
5. We recommend daily use of partially skimmed or skimmed milk or yogurt.
   Milk portion: 150 ml or
   Yogurt portion: 120 g
6. Limit eggs consumption to twice eggs a week.
7. Avoid butter, margarine, lard, palm oil and foods that contain them. Use daily extra virgin olive oil daily. Avoid also fried foods, mayonnaise and pre-cooked frozen food.
   Extra virgin olive oil portion: 3 spoons a day.
8. Eat daily bread, pasta, rice, or other cereals, preferably whole meal. Avoid consumption of fresh egg pasta, oil or milk bread and focaccia.
   Bread portion: 100 g per day (or wholemeal bread: 130 g per day)
   Pasta portion: 80 g per day.
9. Consume legumes at least 2-3 times a week (beans, chickpeas, lentils, peas, broad beans).
10. Consume vegetables and vegetables of any kind daily. Potatoes are not considered vegetables; they are substitutes of bread and pasta and must be consumed no more than once a week due to the high glycemic index.
    Vegetable portion: free.
    Potatoes portion: 350 g instead of 80 g of pasta or 100 g of bread.
11. Consume fruit daily. Limit frequency of consumption of very ripe and sugary fruits (i.e. bananas, persimmons, figs, prickly pears, grapes). Avoid coconut for saturated fat content.

Fruit portion: two per day.
Portion of pineapple oranges, cherries, kiwi, mapo, pears, apples, plums: 150 g
Portion of apricots, watermelon, strawberries, raspberries, quinces, melon, blueberries, blackberries, medlars, peaches, grapefruit: 210 g
Fruits to be limited: bananas, persimmons, figs, prickly pears, mandarins, mangoes, pomegranates, grapes: 100 g

12. Consume a small portion of dried nuts daily (walnuts, almonds, hazelnuts, cashews, pistachios).
Nuts portion: 20 g
13. Sugar should be avoided. Avoid also ice creams, pastry desserts and chocolate consumption. Eat rusks or simple biscuits for breakfast.
Portions: 4 rusks or Wholemeal rusks n.5 or simple biscuits n.4 or bread 40 g.
14. Alcohol consumption is not recommended. Avoid sugar drinks, industrial teas and fruit juices with or without added sugar.
15. 2-3 cups of coffee per day are allowed.
16. Recommended cooking methods: grilled, grilled, steamed, boiled, roasted, in foil, in the oven. To season the dishes, we recommend to use vegetable or meat broth, tomato sauce, vegetables, spices such as basil, oregano, saffron, marjoram, pepper, garlic.
17. Limit use of salt and avoid all foods rich in salt (pickled or canned foods).
18. It is recommended to do regular moderate-intensity exercise (≥ 30 minutes a day)

1.4 General advice based on frequency of food and control of food portions with a total energy intake of 2100 kcal / day for overweight men

1. Limit meat consumption twice a week and prefer white meat (i.e. poultry or lean pork), deprived of skin and visible fat. Limit red meat consumption once a week. Avoid offal consumption (i.e. brain and kidney).
Portion: 150 g
2. Increase fish products consumption at least 2-4 times a week; prefer fish rich in omega-3 fatty acids (sardines, herring, anchovies, salmon). Crustaceans and mollusks are not contraindicated because they contain moderate amounts of cholesterol which are lower than meat and cheese.

	**Portion**
Lobster, crab, shrimp, mussel, squid, cuttlefish, oyster, boga, octopus, sea snails, anchovy, cod, hake, soaked cod, stockfish, sea bream, sole, murmur, snapper, grouper, corvina, dogfish, trout, pagro , croaker, moray, bass, breed, turbot, anglerfish, tench, pike.	200 g
fresh salmon, fresh tuna, carp, mullet, swordfish, mackerel, sardine, herring, sturgeon, sarago, verdesca, latterini, eel.	150 g

3. Limit processed meat consumption once a week and prefer hams deprived of visible fat and turkey. Avoid sausages, bacon, salami and corned beef consumption.
Portion: raw ham 130 g or
cooked ham 150 g or
turkey 190 g.

4. Limit low-fat cheeses and dairy products consumption twice a week. Grated Parmesan should be limited to 1 tablespoon per day.
   Low-fat cheese portion: 80 g
5. We recommend daily use of partially skimmed or skimmed milk or yogurt.
   Milk portion: 200 ml or
   Yogurt portion: 150 g
6. Limit eggs consumption to twice eggs a week.
7. Avoid butter, margarine, lard, palm oil and foods that contain them. Use daily extra virgin olive oil daily. Avoid also fried foods, mayonnaise, and pre-cooked frozen food.
   Extra virgin olive oil portion: 4 spoons a day.
8. Eat daily bread, pasta, rice, or other cereals, preferably whole meal. Avoid consumption of fresh egg pasta, oil or milk bread and focaccia.
   Bread portion: 140 g per day (or wholemeal bread: 180 g per day)
   Pasta portion: 100 g per day.
9. Consume legumes at least 2-3 times a week (beans, chickpeas, lentils, peas, broad beans).
10. Consume vegetables and vegetables of any kind daily. Potatoes are not considered vegetables; they are substitutes of bread and pasta and must be consumed no more than once a week due to the high glycemic index.
    Vegetable portion: free.
    Potatoes portion: 400 g instead of 100 g of pasta or 140 g of bread.
11. Consume fruit daily. Limit frequency of consumption of very ripe and sugary fruits (i.e. bananas, persimmons, figs, prickly pears, grapes). Avoid coconut for saturated fat content.
    Fruit portion: two per day.
    Portion of pineapple oranges, cherries, kiwi, mapo, pears, apples, plums: 150 g
    Portion of apricots, watermelon, strawberries, raspberries, quinces, melon, blueberries, blackberries, medlars, peaches, grapefruit: 210 g
    Fruits to be limited: bananas, persimmons, figs, prickly pears, mandarins, mangoes, pomegranates, grapes: 100 g
12. Consume a small portion of dried nuts daily (walnuts, almonds, hazelnuts, cashews, pistachios).
    Nuts portion: 20 g
13. Sugar should be avoided. Avoid also ice creams, pastry desserts and chocolate consumption. Eat rusks or simple biscuits for breakfast.
    Portions: 6 rusks or wholemeal rusks n.7 or simple biscuits n.5 or bread 70 g.
14. Alcohol consumption is not recommended. Avoid sugar drinks, industrial teas and fruit juices with or without added sugar.
15. 2-3 cups of coffee per day are allowed.
16. Recommended cooking methods: grilled, grilled, steamed, boiled, roasted, in foil, in the oven. To season the dishes, we recommend to use vegetable or meat broth, tomato sauce, vegetables, spices such as basil, oregano, saffron, marjoram, pepper, garlic.
17. Limit use of salt and avoid all foods rich in salt (pickled or canned foods).
18. It is recommended to do regular moderate-intensity exercise (≥ 30 minutes a day)

1.5. Statistical Analysis

Statistical analysis was performed using IBM SPSS Statistics, Version 25.0 (SPSS Inc., Chicago, IL, www.spss.com). A Kolmogorov–Smirnov analysis was performed to test the normality of variables. The results of continuous variables were expressed as median and interquartile range. For ordinal and nominal variables, contingency tables were used for indicating frequency and percentage in the population.

For the comparison of continuous variables between different groups of patients, non-parametric tests of Kruskal–Wallis or Mann–Whitney were used when appropriate. Nominal variables were examined with the Pearson chi square (X^2) test and with Spearman's rank correlation index for the correlation with continuous variables. Intergroup comparisons were adjusted for multiple comparisons with Bonferroni correction. Generalized multivariate model (GLM) was used to adjust the parameters of the lipid profile, considering sex, age, BMI, and smoking habits as fixed factors and covariates.

The evaluation of the differences in the lipid profile in patients according to the consumption levels of different food categories was preliminarily tested with independent sample comparison tests of Kruskal–Wallis or Mann–Whitney when appropriate.

Then, cross-sectional multivariate analyses were performed to reduce the risk of type 2 statistical error (false negative). Multivariate analysis in baseline condition was set as follows: dependent variables were baseline TC, HDL-C, LDL-C, and TG adjusted for sex, age, BMI, and smoking habits and predictors were fruit intake (<150 g/day = 0, 150–300 g/day = 1, and >300 g/day = 2); vegetables intake (<100 g/day = 0, 100–250 g/day =1, and >250 g/day = 2); legumes intake (<70 g/week = 0, 70–140 g/week = 1, and >140 g/week = 2); cereals intake (<130 g/day = 0, 130–200 g/day = 1, and >200 g/day = 2); fish intake (<100 g/week = 0, 100–250 g/week = 1, and >250 g/week = 2); meat products intake (>120 g/day = 0, 80–120 g/day = 1, and <80 g/day = 2); dairy products intake (>270 g/day = 0, 180–270 g/day = 1, and <180 g/day = 2); alcohol consumption (>2 AU/day = 0, 1–2 AU/day = 1, and <1 AU/day = 2); and olive oil use (occasional = 0, frequent = 1, and regular = 2).

Supplementary Table S2. Comedications used by the 106 patients

Treatment	N. of patients
**Antihypertensive drugs [n]**	31
Angiotensin-converting enzyme inhibitors [n]	22
Calcium channel blockers [n]	3
Sartan [n]	5
**Proton pump inhibitors [n]**	11
**D-vitamin [n]**	7
**Levothyroxine [n]**	5
**Lorazepam [n]**	2
**Allopurinol [n]**	1

Supplementary Table S3. Variation of patient distribution according to food categories and the MEDI-LITE score assigned

	**Baseline** [n, %]	**Follow-up** [n, %]	**Variation**[%]	**P-value (Pearson Chi^2)**
**Fruit**				
<150 g/day	15 (20.8%)	7 (9.7%)	-11.1%	<0.0001
150-300 g/day	14 (19.4%)	8 (11.1%)	-8.3%	
>300 g/day	43 (59.7%)	57 (79.2%)	+19.5%	
**Vegetables**				
<100 g/day	23 (31.9%)	12 (16.7%)	-15.2%	<0.0001
100-250 g/day	21 (29.2%)	17 (23.6%)	-5.6%	
>250 g/day	28 (38.9%)	43 (59.7%)	+20.8%	
**Legumes**				
<70 g/week	37 (51.4%)	16 (22.2%)	-29.2%	<0.0001
70-140 g/week	26 (36.1%)	24 (33.3%)	-2.8%	
>140 g/week	9 (12.5%)	32 (44.4%)	+31.9%	
**Cereals**				
<130 g/day	25 (34.7%)	9 (12.5%)	-22.2%	0.324
130-200 g/day	15 (20.8%)	17 (23.6%)	+2.8%	
>200 g/day	32 (44.4%)	46 (63.9%)	+19.5%	
**Fish**				
<100 g/week	21 (29.2%)	3 (4.2%)	-25.0%	0.010
100-250 g/week	39 (54.2%)	25 (34.7%)	-19.5%	
>250 g/week	12 (16.7%)	44 (61.1%)	+44.4%	
**Meat Products**				
>120 g/day	17 (23.6%)	7 (9.7%)	-13.9%	0.009
80-120 g/day	19 (26.4%)	12 (16.7%)	-9.7%	
<80 g/day	36 (50.0%)	53 (73.6%)	+23.6%	
**Dairy Products**				
>270 g/day	37 (51.4%)	15 (20.8%)	-30.6%	0.001
180-270 g/day	6 (8.3%)	31 (43.1%)	+34.8%	
<180 g/day	29 (40.3%)	26 (36.1%)	-4.2%	
**Alcohol**				
>2 AU/day	28 (38.9%)	12 (16.7%)	-22.2%	<0.0001
1-2 AU/day	20 (27.8%)	28 (38.9%)	+11.1%	
<1 AU/day	24 (33.3%)	32 (44.4%)	+11.1%	
**Olive Oil**				
Occasional	1 (1.4%)	0 (0%)	-1.4%	0.883
Frequent	3 (4.2%)	4 (5.6%)	+1.4%	
Regular	68 (94.4%)	68 (94.4%)	+0.0%	

Abbreviations: AU = Alcoholic Unit.
